# Needs and Expectations Associated With an e–Mental Health Intervention for Reducing Somatoform, Anxiety, and Depressive Symptoms in Sexual and Gender Minority Adults: Qualitative Participative Study

**DOI:** 10.2196/65834

**Published:** 2025-09-12

**Authors:** Anna Gomes, Angeliki Tsiouris, Florian Jung, Laura Rebecca Klein, Adina Kreis, Manfred E Beutel, Bernhard Strauss, Madita Hoy, Rüdiger Zwerenz

**Affiliations:** 1 Department for Psychosomatic Medicine and Psychotherapy University Medical Center of the Johannes Gutenberg-University Mainz Mainz Germany; 2 Institute of Psychosocial Medicine, Psychotherapy and Psychooncology University Hospital Jena Jena Germany

**Keywords:** sexual orientation, gender identity, sexual and gender minority, minority stress, qualitative methods, prevention, barriers to care, e–mental health, community-based participatory research

## Abstract

**Background:**

Sexual and gender minority individuals experience heightened risks of mental health disorders due to marginalization, discrimination, and inadequacies in health care.

**Objective:**

This study aims to identify the needs and expectations concerning an e–mental health intervention designed for people who are lesbian, gay, bisexual, transgender, queer or questioning, intersex, asexual, or have other sexual orientation and gender identities (LGBTQIA+) to reduce somatoform, anxiety and depressive (SAD) symptoms.

**Methods:**

A qualitative participative study was conducted, involving semistructured interviews (face-to-face and online) with 10 sexual and gender minority individuals experiencing SAD symptoms. Telephone interviews were conducted with 10 health care professionals (HCPs). This study was part of a participatory project, emphasizing cooperation with the LGBTQIA+ community. Data were analyzed through a deductive-inductive content analysis to derive categories of needs and expectations relevant for the development of an e–mental health intervention.

**Results:**

Participants expressed a strong desire for the intervention to be inclusive, validating, and sensitive to the unique challenges faced by LGBTQIA+ people. Key themes included the need for information on the relationship between being queer and mental health; representation through case stories; psychoeducation; and exercises tailored to address minority stress, identity affirmation, and coping strategies. HCPs emphasized the importance of addressing the coming-out process, managing rejection, fostering self-acceptance, and including content on minority stress and its impact on mental health. Results of both interview groups highlighted the need for direct interaction with therapists or peer support, including both synchronous and asynchronous elements (eg, video calls and chat) based on nonheteronormative, sensitive therapeutic support, for example, avoiding preassumptions, using sensitive language, and reflecting possible trigger points.

**Conclusions:**

This study underscores the need for e–mental health interventions tailored to a queer-sensitive and participatory approach. Interventions should incorporate comprehensive psychoeducation, interactive elements, content reflecting the lived experiences of LGBTQIA+ individuals with SAD symptoms, and the possibility to connect and exchange experiences with others facing similar challenges. Engaging with both LGBTQIA+ people and HCPs in the development process is essential to ensure the intervention’s relevance, effectiveness, and acceptability.

## Introduction

### Theoretical Background

Sexual and gender minority (SGM) individuals, encompassing nonheterosexual, transgender, and nonbinary populations, represent a demographic group that has historically experienced marginalization, mistreatment, pathologization, and neglect within a predominant cis-heteronormative society [1,2]. Existing inadequacies, such as microaggressions, are also barriers to health care for individuals who are lesbian, gay, bisexual, transgender, queer or questioning, intersex, asexual, or have other sexual orientation and gender identities (LGBTQIA+) [3-6]. Looking at LGBTQIA+ individuals with mental burden, it is important to acknowledge that identifying as an SGM individual is often associated with specific mental health risk factors that might not apply to cisgender heterosexual individuals [7,8]. The theory of minority stress [9] postulates that belonging to a minority group might be connected with distal factors, such as discrimination and victimization experiences, and proximal factors, such as fear of rejection, insecurity within the system, and internalized negative attitudes toward one's own identity [10]. Hence, health-related stereotype threats play a major role among SGM individuals and have a strong influence on their behavior. They are often associated with fear of physicians, delays in seeking mental health services, and poor self-reported mental health outcomes [11,12]. Experiencing stigma and discrimination elevates distress and limits effective coping strategies that interfere with positive development, health, and well-being in SGM individuals [13]. This is critical, as the currently limited empirical research on SGM individuals in Germany already shows a markedly increased risk of mental health disorders within this group. The risk of experiencing depression or burnout, for instance, is 3 times higher for members of the LGBTQIA+ community compared to the general population [14]. International research shows that lesbian, gay, bisexual, and transgender (LGBT) adolescents are 1.75 times more likely to experience symptoms of anxiety and depression than their cisgender heterosexual peers [15]. Finally, research suggests a higher risk for suicidal behaviors among LGBT populations [16]. Besides internalized homophobia and shame, significantly poorer mental health outcomes were identified as risk factors for suicide in LGBT populations [17].

E–mental health interventions promise effective ways to bridge treatment gaps [18-20]. Due to the described experiences within the health care system, many SGM individuals have a high affinity for online platforms to seek health-related information and support [21]. Internationally, a few clinical trials evaluated LGBTQIA+-specific e–mental health interventions with promising results. In the United States, a randomized controlled trial evaluating an online intervention revealed that LGBTQIA+ participants allocated to an expressive writing group or a self-affirmation intervention reported significant positive changes in several mental health parameters compared with participants in the control group [22]. In Germany, a treatment tailored to the needs of LGBTQIA+ individuals with mental disorder symptoms is not yet available. To the best of our knowledge, existing German e–mental health interventions mostly represent hetero-normative life realities in their content, so that persons outside these life realities often feel not sufficiently reflected. However, evidence is scarce, and research on the specific needs of LGBTQIA+ individuals regarding the content of an e–mental health intervention is crucial in order to develop an intervention that is effective in reducing symptoms of mental disorders and does not exclusively reproduce cis-heteronormative content.

In the development of online interventions, it is recommended to involve end users in the design process, a practice commonly applied in approaches such as user-centered design and cocreation. It leads to increased adherence and effectiveness of the intervention and is hence state of the art in intervention development [23,24]. Although there is consensus on the necessity of involving end users, various approaches with different levels of participation do exist [25,26]. For individuals who frequently encounter experiences of discrimination and marginalization, a high level of participation in the development has an even greater significance [27]. This guarantees an empowerment-oriented, sensitive approach with communication in all phases of research and development.

It is thus considered pivotal, in a first step, to capture the unmet needs and expectations of LGBTQIA+ people regarding an online intervention to ensure that the intervention aligns with the target group’s needs. Using a participatory approach even at this stage could also lead to participants feeling welcome, validated, and understood—an experience that many LGBTQIA+ adults often lack not only in their social environments but also in the medical and therapeutic context.

### Research Objectives

We do not know of any previous participative research studies that have investigated LGBTQIA+ individuals’ expectations and needs regarding an e–mental health intervention. This study is part of the Queer-E–Mental Health (Queer-EMH) project, a bicentric research project carried out by the University of Mainz and the University of Jena that aims to design an effective e–mental health intervention for LGBTQIA+ individuals with mental burden in a participatory study design and establish an LGBTQIA+ advisory board. To tailor this intervention specifically to the needs of the target group, their specific needs and expectations were investigated. In terms of a user-centered development process, this study’s objectives are as follows:

1. To determine attitudes, expectations, and potential barriers associated with psychological online interventions aiming at reducing somatoform, anxiety, depressive (SAD) symptoms in LGBTQIA+ individuals with mental burden
2. To obtain information and recommendations from health care professionals (HCPs) and counseling staff with experience in working with LGBTQIA+ people about what an e–mental health intervention should contain and how it should be designed to reach the target group

## Methods

### Research Team and Reflexivity

This project was supported by an LGBTQIA+ advisory board, which was established at the beginning of the project and consisted of members of 10 German LGBTQIA+ associations. In a participatory setting, the LGBTQIA+ advisory board ensured that the study processes proceeded in a participant-friendly and queer-sensitive manner. In numerous sessions, work packages in the project (eg, recruitment procedures, wording of recruitment materials, development of questions for the interview guide, and data interpretation) were collaboratively developed and worked on.

One of the interviewers simultaneously worked as a coder. The interviews were conducted by researchers AT, AG, and Clara Pering at the study center in Mainz and by MH at the study center in Jena. All interviewers were female. No personal information about the interviewers (such as sexual orientation) was revealed to participants. There were no personal relationships between the interviewers and participants before the study. As clinical psychologists and experienced psychotherapy researchers, the interviewers are trained in qualitative research methods and the clinical picture of SAD disorders. Through their collaboration with our advisory board, they gained essential insights into relevant aspects of queer identity and living environments. A personal queer biography was not a prerequisite for the interviewers.

### Ethical Considerations

Ethics approval for the study was obtained from the ethics committee of the Medical Association of the Federal State of Rhineland-Palatinate (registration number 2022-16876_1-NIS) and the ethics committee of the Jena University Hospital (registration number 2022-2855-Bef). All participants gave their informed consent on paper and were informed that participation was voluntary and that they could withdraw their consent at any time. The data were pseudonymized. Personal contact details of the participants were stored separately from interview data, inaccessible to third parties. There were no financial incentives for participants in this study. This study followed the COREQ (Consolidated Criteria for Reporting Qualitative Research) guidelines (Multimedia Appendix 1).

### Recruitment and Participants

We conducted a qualitative study, with data collected via semistructured interviews with LGBTQIA+ individuals and telephone interviews with HCPs and counseling staff who regularly worked with LGBTQIA+ individuals with SAD symptoms (eg, consultation or psychotherapy). Using a cocreative, participatory approach, we ensured that the conceptualization of a queer-sensitive EMH intervention addresses the actual challenges perceived not only by LGBTQIA+ individuals but also by HCPs and counseling experts.

We recruited LGBTQIA+ individuals using various recruitment strategies. A central pillar of the recruitment was the Instagram project account (Meta Platforms, Inc) on which we published information about our study and referred to the possibility of participating. Besides social media recruitment, the cooperating LGBTQIA+ advisory board representatives distributed the study flyer and issued the call for participants within their networks (mainly via email). The study flyer contained information on the study procedure, inclusion criteria, and contact details for the study staff. Participants were included if they fulfilled the following inclusion criteria: (1) identified as LGBTQIA+ individuals, (2) reported SAD symptoms (currently or in the past), (3) were aged ≥18 years, (4) had sufficient German language skills to participate in an interview or focus group, and (5) were able to provide informed consent. Participants were excluded if they had severe mental burden (eg, severe depression and suicidal tendencies). In this case, the study staff would provide information on where to find professional support.

HCPs were recruited with the support of the LGBTQIA+ advisory board. HCPs interested in study participation reached out to the project team and were enrolled if they met the following inclusion criteria: (1) had experience counseling LGBTQIA+ individuals with mental disorders, (2) were aged ≥18 years, (3) had sufficient German language skills, and (4) provided informed consent.

### Study Procedure

Interested individuals contacted the study team and were enrolled in the study following a telephone screening. During the telephone screening, participants were screened for the inclusion and exclusion criteria and informed about the aim and potential risks of the study (ie, exposure to topics that might trigger an emotional reaction, such as sadness, anxiety, or anger). With regard to symptomatology, participants were asked a yes or no question during the phone call about whether they were currently experiencing or had previously experienced SAD symptoms. Thus, participants self-referred to either the interview or 1 of the 4 focus groups that took place during the same time period. In this paper, we analyzed the interview results only.

A total of 51 interested persons contacted the research staff. We included participants in our study consecutively based on the date on which participants expressed their interest in the study. In addition, we took care to ensure representation of a diverse range of gender identities and sexual orientations among the participants, aiming to incorporate various subgroups within the LGBTQIA+ community into the study. One participant was excluded from the analyses of this study, as attitudes regarding online interventions were not discussed because the participant had already participated in the focus group earlier and provided feedback regarding the relevant questions in the focus groups. We stopped including participants after reaching the target of 10 participants. Because the qualitative study presented in this paper was only a small subproject within a larger project aimed at developing a concept for the corresponding intervention, the available personnel and time resources were limited, leading to the predetermined restriction on the number of interviews.

The interviews were conducted in a 2-person setting, either face-to-face in the study centers (Mainz and Jena) or online via a secure video platform. All interviews were audio recorded and subsequently transcribed and coded using MAXQDA (version 11.1.4; VERBI Software).

### Interview Guide

The semistructured interview guide was developed in an iterative process in close cooperation with representatives of the LGBTQIA+ advisory board. In detail, the research staff collected relevant research topics and proposed specific questions and prompts, which were then presented to the advisory board and discussed in regard to their relevance and appropriateness (eg, in terms of sensitive language and potential triggers). The feedback process was repeated until the research staff and the representatives of the LGBTQIA+ advisory board agreed on the interview guide.

The interview started with an open-ended question about the participants’ motivation to contribute to the study, followed by items querying the main topics (1) subjective mental illness concept, (2) medical and psychotherapeutic experiences, (3) attitudes and expectations toward e–mental health interventions, and (4) personal strengths and conclusion. While the primary objective of the interviews was to gather information on topic 3, the detailed introduction served to provide participants with time to ease into the conversation and establish a validating, understanding atmosphere from the outset, encompassing feelings and difficult experiences within and beyond the health care system. This was particularly crucial, as the interviewers, working in a clinic, were inherently part of the health care system, necessitating the dismantling of any preexisting reservations.

In this study, we analyzed and interpreted participants’ responses to the main topic 3. The interview questions querying attitudes and expectations toward a queer sensitive online intervention are reported in Textbox 1. While this study examined not only content-related expectations but also aspects of presentation format and structural considerations, this paper focuses on presenting and discussing the findings related to content expectations. This decision was driven by the fact that presentation and structural requirements (eg, mode of delivery) did not differ significantly from those of other target groups of online interventions and have already been extensively researched, with a wealth of existing literature covering these aspects comprehensively [28,29].

**Interview questions querying attitudes and expectations of individuals who are lesbian, gay, bisexual, transgender, queer or questioning, intersex, asexual, or have other sexual orientations and gender identities (LGBTQIA+) toward a queer-sensitive online intervention.**

**Needs and expectations**

- What would you like to see in an e–mental health intervention tailored to the needs of the LGBTQIA+ community?
- What are your expectations for an e–mental health intervention tailored to the needs of the LGBTQIA+ community?
- What advantages and disadvantages or caveats might be associated with an e–mental health intervention for members of the LGBTQIA+ community?

Qualitative data from the interviews were supplemented by sociodemographic data collected with a brief questionnaire, which was filled out by each participant before the interview started. The short questionnaire assessed gender identity, sexual orientation, year of birth, place of residence, marital or relationship status, highest education, and whether participants were currently on sick leave.

### Qualitative Content Analysis

The coding guide was developed following an iterative deductive-inductive procedure by first deriving relevant categories from the literature (deductive) and then adding additional categories based on the data collected in the interviews (inductive). Each interview was coded by 2 persons and ratings were compared in terms of consensus. Deviating codes were discussed until consensus was reached. Frequencies of statements in the respective main and subcategories were analyzed descriptively. During the data analysis, the results were reviewed and interpreted together, with the LGBTQIA+ advisory board providing valuable support in contextualizing quotes, which added significant value to the interpretation of the findings.

### Telephone Interviews With HCPs and Counseling Staff

In order to supplement attitudes toward and expectations from an e–mental health intervention from the perspective of LGBTQIA+ persons with symptoms of mental health disorders, we also conducted structured telephone interviews with 11 HCPs. The structured interview guide was developed in close cooperation with the LGBTQIA+ advisory board. The interview guide consisted of the following main topics: (1) perceived treatment needs of LGBTQIA+ individuals with SAD disorders, (2) LGBTQIA+ individuals in the German health care system, and (3) attitudes toward e–mental health interventions for LGBTQIA+ individuals. Similar to the interviews with LGBTQIA+ individuals, this paper analyzes responses by HCPs and counseling staff to the main topic 3. Detailed questions are presented in Textbox 2.

Interviews with HCPs were conducted by telephone and systematically documented in written bullet points by the interviewers. These bullet points were then transferred into the software MAXQDA and coded into categories. The qualitative content analysis of the data assessed in the telephone interviews with HCPs and counseling staff followed the same deductive-inductive approach and procedure as the data assessed in the face-to-face interviews.

**Interview questions querying the expectations and concerns of health care professionals and counseling staff regarding an online intervention for individuals who are lesbian, gay, bisexual, transgender, queer or questioning, intersex, asexual, or have other sexual orientations and gender identities (LGBTQIA+).**

**Needs and expectations**

- What characteristics would an online intervention for LGBTQIA+ people with mental illness need to have for you to recommend it?
- What benefits might an online intervention provide for LGBTQIA+ people with mental illness?

**Concerns and barriers**

- What concerns do you personally have about e–mental health interventions?
- What concerns do you think your clients might have about e–mental health interventions?

## Results

### Research Objective 1: The Perspectives of LGBTQIA+ Individuals

#### Overview

The final sample consisted of 10 adults with symptoms of SAD disorders who identified themselves as LGBTQIA+. Table 1 displays sample characteristics. Participants were aged between 23 and 67 (mean 38.2, SD 12.75) years. Half (n=5, 50%) of the participants were single and had a diploma qualifying for university. Most (n=6, 60%) of the participants were currently on sick leave or retired. The interviews lasted for an average duration of 72.8 (SD 11.35) minutes.

**Table 1:** Sociodemographic characteristics of the sample of individuals who are lesbian, gay, bisexual, transgender, queer or questioning, intersex, asexual, or have other sexual orientations and gender identities (N=10).

Variable			Values
Age (y), mean (SD)			38.2 (12.75)
**Gender or gender identity, n (%)**			
	Cisgender female	2 (20)	
	Cisgender male	1 (10)	
	Nonbinary and transmasculine	1 (10)	
	Nonbinary	2 (20)	
	Transgender female	3 (30)	
	Intersex female	1 (10)	
**Sexual and romantic orientation, n (%)**			
	Gay	2 (20)	
	Lesbian	2 (20)	
	Biromantic and asexual	1 (10)	
	Bisexual	1 (10)	
	Demisexual	1 (10)	
	Pansexual	1 (10)	
	Queer (pansexual)	1 (10)	
	Individual	1 (10)	
**Current relationship status, n (%)**			
	Single	5 (50)	
	In a relationship	3 (30)	
	Married or civil union	2 (20)	
**Highest education, n (%)**			
	No degree or lower secondary education diploma	3 (30)	
	General secondary education diploma	1 (10)	
	Diploma qualifying for university	5 (50)	
	University degree	1 (10)	
Currently on sick leave			4 (40)
Retired			2 (20)

**Figure 1:**
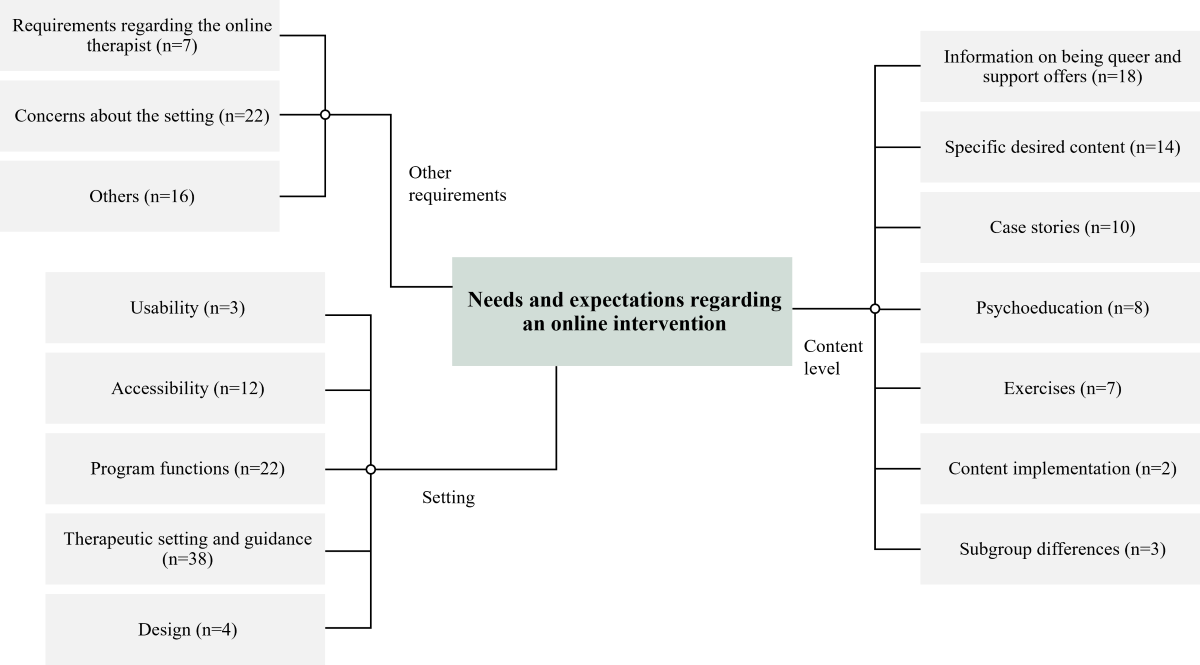
Number of codes by category and subcategory in the qualitative content analysis of the interviews with individuals who are lesbian, gay, bisexual, transgender, queer or questioning, intersex, asexual, or have other sexual orientations and gender identities with symptoms of depression, anxiety, or somatoform disorders.

The previously described inductive-deductive procedure revealed 4 broad categories. Each category described a different level into which the expectations, barriers, and requirements regarding an online intervention for LGBTQIA+ individuals with SAD disorders, as raised in the interviews, could be classified: (1) content level, (2) linguistic level, (3) framework, and (4) other requirements. Figure 1 provides an overview of the number of codes assigned to the respective categories and subcategories. The subsequent paragraph presents participants’ experiences categorized by those themes. We have put a special focus on the presentation of the category *content level*. The 3 remaining categories (linguistic level, setting, and other requirements) are subsequently briefly presented, and characteristics specific to the LGBTQIA+ population are highlighted. We have then presented complementary data drawn from the HCPs’ interviews, highlighting similarities and differences in their respective perspectives.

#### Content Level

##### Information About Being Queer and Support Offers (18 Codes)

On the basis of the feedback from most (7/10, 70%) of the participants, it became clear that integrating comprehensive information resources on queerness within the planned online self-help intervention is crucial. While the internet offers a wealth of information, distinguishing credible sources remains a challenge. For example, certain online content, such as definitions of specific terms, was reported as being discriminatory and emotionally harmful:

Because I...have my information since I was 14, that’s when it started, that I collected information, but it must also be said that this collection has led to a lot of harmful contacts on the internet with harmful material, which is sexist, objectifying or simply represents hate crime somewhere. And I think that a self-help service like this, where all the information is brought together in written form with experiences, would be very helpful.

In addition to the limited resources addressing critical topics, such as the impacts of hormone treatments during transitioning, participants reported a lack of easily accessible avenues for locating practical assistance. Participants expressed the need for a comprehensive directory of LGBTQIA+ friendly contacts, counseling services, support networks, outpatient facilities, and affirming medical practitioners and reported that this dearth of resources often perpetuates feelings of helplessness and isolation:

I was misgendered, I was not respected, I was not accepted. How do I deal with that? Where do I go for help? Where do I go for support? To put it bluntly, where can I have a good cry? Where can I get hope?

##### Specific Desired Content (14 Codes)

A wide variety of content-related specific wishes and ideas was expressed by the participants (4/10, 40%), summarized under the category *specific desired content*. Significant attention revolved around the basic attitude with which the program and content should be designed. The participants expressed a deep desire to feel seen and acknowledged within the program. In this context, they referred to the predominantly hetero- and cis-normative society, in which they perceived a lack of acknowledgment and representation of LGBTQIA+ persons. The desire for validation and acceptance of one’s own gender identity and sexual orientation as well as the normalization of the full spectrum of emotions associated with this aspect of personal identity was an important aspect that was repeatedly emphasized in all interviews. In addition, participants expressed strong interest in understanding the intersection between mental health challenges and the experience of being queer, including insights into how minority stress could potentially contribute to mental health disorders. Concerning therapeutic content, a recurring theme among many participants was to address their own emotions within the online intervention, with a focus on exploring, validating, and normalizing those feelings:

Include the emotional more....Emotions play a crucial role here, especially when it comes to one’s own gender or sexual identity. This is inextricably linked with emotions. And to give space to the emotions and to confirm them...it is okay, if you are afraid of coming out, for example, or if you are angry that you are going through this experience, that it makes you sad, that is all valid at first. And then: That’s where you can continue to work, so it’s not like you’re caught up in the emotions all the time, but you can work with that.

There were also statements that related specifically to the therapeutic rationale. For example, one participant demanded clear strategies and tools, while another participant emphasized their need for the therapeutic orientation to go beyond standard psychotherapeutic approaches, such as cognitive behavioral therapy (CBT), which is currently most frequently used in the online context:

I think what would be important for me, for example, is not only to go to CBT, as useful as that is, but that doesn’t work for every person. And maybe also to create offers, if that is somehow possible, that you can also control a little bit in which direction you want to go or what helps you. Because I often have the feeling that such offers are often very CBT-based. And as helpful as some exercises and some things are for me, I...often have the feeling...that often doesn’t really work for me.

##### Case Stories (10 Codes)

Many (4/10, 40%) interviewees expressed the desire to integrate personal case examples featuring individuals representing diverse life realities into the online intervention:

Or for example “I am gay. I came out at home. My parents kicked me out.” Unfortunately, that still happens very often today. Where do I go then if I can’t go to friends or family? And then that person just tells how they dealt with it. What did she do, where did she go? Did she go to friends’ houses first, have a good cry, sleep on the couch or something? Or did she go to some counseling center and find someone to stay with? And when that is told as a personal experience, it gives you a bit of courage. And gives orientation.

Furthermore, in the context of dealing with complex emotional responses, particularly regarding self-discovery and identity formation, the central role of these role models in normalizing a broad spectrum of intense emotions was underscored:

I find it helpful when other people describe their own emotions and experiences. Above all, that, this emotional aspect also comes into it, because of course finding one’s identity is connected with a lot of emotion. That this is really described a bit...That you realize that you are not alone with these emotions. That you feel represented and also seen.

##### Psychoeducation (8 Codes)

Concerning the participants’ mental distress, a need for psychoeducation on mental health disorders was evident (6/10, 60%). This included not only information on the nature of mental illnesses, for example, their development, progression, associated symptoms, maintaining factors, and explanatory models, but also comprised the role of emotions, the interplay between mind and body, and issues such as suicidality. In addition, the intersections between mental symptomatology and being queer were highlighted as crucial points of interest:

I am currently thinking about whether it would be useful, if such an online offer also had short explanations about anxiety disorders, for example, social anxiety, such as a graphic, as my therapist drew, of how social anxiety develops,... perhaps that could help....

It was emphasized several times that while it is important to address and explain connections between mental distress and being queer, it is also crucial to make a clear distinction between mental health conditions and sexual or romantic orientation or gender identity. This referred especially to the fact that, in any case, the impression that being an individual belonging to the LGBTQIA+ community would be equated with mental illness should be avoided, as this, in turn, would reproduce often experienced stigmatizations and prejudices:

[S]o to clearly separate sexual or romantic orientation and the clinical syndromes, because that blurs a lot. To be safe,...there is nothing wrong with me in terms of health.

##### Exercises (7 Codes)

Specific suggestions for possible exercises were also raised in the interviews by the participants (5/10, 50%), mostly taken from personal experiences that the interviewees had and considered helpful. In addition to frequently mentioned mindfulness and relaxation exercises, gratitude exercises and questions reflecting oneself and one’s own past were suggested. Another interviewed participant emphasized the importance of open-ended questions, which can provide an impulse to reflect on personal issues and thereby gain insights for oneself:

[O]r relaxation techniques to switch off and no longer be trapped in this circle of thoughts, concrete things that are also easily accessible and where you can sit down briefly and say, “I’m now doing the meditation or the exercise.”

##### Subgroup Differences (3 Codes)

As it was one of the study’s objectives to determine whether it is realistic and feasible to develop a single intervention targeting the entire LGBTQIA+ community with SAD disorders or specific content is needed for different subgroups (eg, gender identity vs sexual orientation), these questions were also explored in the participant interviews (3/10, 30%). Surprisingly, few relevant differences between subgroups emerged. The differences mentioned were only related to information needs. It was noted that, for example, transgender individuals will benefit from information about the transition process, whereas this information is not relevant for cisgender individuals, and appropriate customization options should be available in the online intervention. Given the approach that the goal of the online intervention was to reduce psychological symptoms, the project was not considered too ambitious or unrealistic. Psychological symptoms related to minority stress (eg, arising through the feeling of being different) were always highlighted as a unifying element:

Then every special path is of course different...whether it is homosexuality or trans is a huge difference and to have a gender matched surgically—there are worlds in between.... But first of all, this common basic thing I can picture well: How do you deal with it when you have the feeling...I am different?

#### Setting and Other Requirements

Table 2 presents a comprehensive summary of the principal components classified under the 2 remaining primary categories, namely *other requirements* and *setting*. It is noteworthy that there was a strong emphasis on the importance of accessibility concerning the way of delivering e–mental health interventions. In addition, there was a frequent desire for direct interaction with a therapist, including both synchronous (eg, video calls) and asynchronous methods (eg, chat). None of the participants stated that they did not want to use an online intervention due to concerns about data security and anonymity.

**Table 2:** Key aspects related to the setting and other requirements for an online intervention for individuals who are lesbian, gay, bisexual, transgender, queer or questioning, intersex, asexual, or have other sexual orientations and gender identities with symptoms of depression, anxiety, or somatoform disorders, as identified in the qualitative interviews.

Category and subcategory^a^			Results
**Setting**			
	Therapeutic setting and guidance (n=38)	Desire for direct interaction with therapists or contact persons through video or chat	
	Program functions (n=22)	Frequent requests for the opportunity to connect with others, for example, through a forum or mentorship program Requirement to maintain a clear individualized path, for example, with graphical elements	
	Accessibility (n=12)	Importance of maintaining a low threshold and good program accessibility, such as providing free access due to financial disparities, considering neurodivergent individuals or people with disabilities Preservation of participant anonymity as a crucial component	
	Design (n=4)	Emphasis on a modern and professional design Inclusion of elements reflecting diversity Preference for a minimalistic design	
	Usability (n=3)	Requirement for a simple and clearly structured user interface	
**Other requirements**			
	Concerns about the setting (n=22)	Concerns regarding the preservation of participant anonymity and highlighting the need for a safe space Apprehensions about the lack of alignment with real-life situations Need for the introduction of novel insights; avoidance of reiteration of already-known information	
	Requirements regarding the online therapist (in case of a guided intervention; n=7)	High qualifications of online therapists Taking a non–cis-heteronormative approach in therapy Proficiency in queer issues and familiarity with the associated terminology (ie, inclusive, validating, and sensitive language use; awareness for preassumptions; and reflecting trigger points)	
	Others (n=16)	Clear management of participant expectations, detailing program capabilities and limitations Consideration of comorbidities in therapy planning Guidance on the process of finding suitable therapy providers	

^a^The numbers in parentheses indicate the absolute number of codes in each subcategory.

### Research Objective 2: The Perspective of HCPs and Counseling Staff

This analysis focused exclusively on the aspects categorized under the *content level* section, as these elements are of particular significance to the scope of this paper. Table 3 displays sample characteristics.

**Table 3:** Sociodemographic characteristics of the health care professionals (N=10).

Variable		Values
Age (y), mean (SD)		39.7 (8.75)
**Gender and gender identity, n (%)**		
	Cisgender female	2 (20)
	Cisgender male	3 (30)
	Nonbinary and transmasculine	1 (10)
	Nonbinary	1 (10)
	Transgender male	2 (20)
	Intersex female	1 (10)
**Sexual and romantic orientation, n (%)**		
	Gay	3 (30)
	Bisexual	1 (10)
	Heterosexual	1 (10)
	Homosexual and panromantic	1 (10)
	Androphile	1 (10)
	Pansexual and polyamorous	1 (10)
	Queer (all genders)	1 (10)
	Other	1 (10)
**Occupational status, n (%)**		
	Employed	6 (60)
	Civil servant and self-employed	2 (20)
	Employed and self-employed	2 (20)

Across all subcategories, HCPs provided significantly more content-related suggestions. They proposed specific exercises and introduced therapeutic topics that should be considered in the online intervention.

With regard to *information on queer identity and support services* (16 codes; 6/10, 60%), HCPs commonly emphasized the significance of the minority stress model [8,9] in the conception of content for the online intervention. In addition, from the HCPs’ perspective, information should be provided on learned internalized hostilities, shame, doubts, and uncertainties in dealing with one’s own sexual or gender orientation. Many topics previously raised by patients were also mentioned by the HCPs, such as clarification of the connection between queer identity, being stereotyped through othering and mental health, psychoeducation, and the presentation of networking opportunities. One topic not previously mentioned by patients but by HCPs was the discussion of attachment and attachment traumas, as this was perceived as important in counseling and psychotherapy.

Specific desired contents (32 codes; 10/10, 100%) of the intervention, according to the HCPs, primarily focused on managing the coming-out process (both within immediate family and in work or other social contexts) and dealing with the rejection of close family members and the associated emotional injuries. Other desired content included self-reflection on one’s life trajectory and significant life events, a general examination of one’s social relationships (including reflection on one’s role within the family system), and coping with the ongoing conflict between closeness and distance. Similar to the patients, HCPs also frequently addressed coping with issues such as bullying, discrimination, and loneliness.

While patients expressed a strong desire for *case stories*, this aspect was less prominent in the HCP (3 codes; 3/10, 30%) interviews. Although the HCPs did mention the use of personal experiences or case examples as a potential means to demonstrate to participants that they are not alone and illustrate the possibility of corrective experiences, this topic held less prominence compared to the patient interviews.

All interviewed HCPs (10/10, 100%) placed a great emphasis on the category of *psychoeducation* (31 codes) and mentioned a large number of relevant points. In addition to the topics frequently mentioned by both patients and HCPs (eg, bio-psychosocial explanatory model and explanation of mental health disorders and their development), physicality and connections between body and psyche were particularly emphasized from the HCPs’ point of view.

Compared to the patients, the HCPs reported significantly more concrete ideas of suitable *exercises* (19 codes; 7/10, 70%) which, in their view, could be integrated into the online intervention. The exercises mentioned included exercises not only on self-esteem and self-acceptance but also social skills training, resilience, and skills and self-determination in the transition process.

In view of *differences among the various subgroups within the community* (9 codes; 8/10, 80%), the group of intersex individuals and the group of transgender persons were mentioned as subgroups that could potentially differ from the other groups in terms of their needs for the planned online intervention.

Regarding *language* (16 codes; 6/10, 60%), HCPs stressed the need for the language used in the online intervention to be both considerate and mindful of gender sensitivity. The users’ identities and orientations should be respected, and this inclusivity should be reflected in the language, exemplified by the respectful use of preferred pronouns. In addition, the language should avoid excessive medical jargon or unnecessary complexity. The correct use of terminology related to specific LGBTQIA+ issues was also considered crucial.

## Discussion

### Principal Findings

As outlined by Pachankis [30], there is a pronounced need for studies to investigate whether and how existing evidence-based interventions need to be adapted to provide a queer-sensitive e–mental health intervention. To the best of our knowledge, this participative study is the first to provide insights into the specific needs, from both a patient’s and HCP’s perspective, regarding an online intervention aimed at improving mental health in LGBTQIA+ adults. This is an important prerequisite for developing a specific online intervention to reach the marginalized target group and address the supply gap.

In the interviews, across all categories, a clear desire became evident to be represented, seen, and accepted and to feel good about oneself with one’s gender identity and sexual orientation. This is consistent with scientific findings indicating that SGM individuals frequently encounter discriminatory experiences in their daily lives and within the health care system [6]. It also aligns with recent research highlighting that aware, sensitive behaviors as well as community-oriented strategies are key resilience factors for transgender, nonbinary, and gender nonconforming individuals in coping with minority-stress [31].

In line with the minority stress model [9], where social support is considered a significant protective factor against negative mental effects of minority stress, our study also revealed a desire to connect and exchange experiences with others facing similar challenges. Studies also demonstrate the significant importance of engaging with others when it comes to the mental health of SGM groups. For example, a systematic review by Valentine and Shipherd [32] demonstrated that social support was central in protecting against mental health distress among transgender and gender nonconforming people in the United States, and the absence of social support was associated with poor outcomes. A study by Jacmin-Park et al [33] revealed that during the COVID-19 pandemic, social support networks available for SGM individuals had a buffering effect against depression that was 4 times stronger than for cisgender heterosexual individuals. They concluded that fostering connections and supporting the development of so-called “families of choice” appeared to be even more significant for SGM individuals than for cisgender heterosexual individuals in coping with crisis periods. This is presumably because SGM individuals more frequently encounter challenging and discriminatory experiences in their everyday interpersonal relationships. The internet makes it easy to contact other people, so it is not surprising that, for example, social media can hold particular significance for SGM youth and serve as a space where they can rely on a supportive online community and develop a positive SGM identity [34]. From this perspective, it is crucial, especially in the development of an online platform for SGM individuals, to offer a means of mutual exchange.

A significant topic that emerged from the interviews, which has received relatively little attention in the literature so far, is the desire for an online resource providing clear and credible information. The interviewed individuals expressed a wish for information not only about being queer itself and related topics (eg, information about transition processes) but also about mental health in general (eg, the development of depression) and the connections between these 2 areas (eg, the minority stress model). Individuals sometimes feel overwhelmed during online searches or may even encounter harmful content reproducing discriminatory experiences. The results are in line with a study in which interviews were conducted with SGM adolescents experiencing at least mild anxiety or depressive symptoms, aiming to explore how online mental health help-seeking interventions could be designed [35]. Among the interviewed adolescents, a strong need for informational resources became evident. When considering the results of our study, it is noteworthy that this need persists into adulthood and does not seem to be limited to adolescents.

The overall results align with the findings by Steinke et al [36], who investigated the needs of SGM youth regarding digital health interventions through interviews and focus groups. Their study highlights the desire for finding a supportive and validating environment and connecting with others who share similar experiences and the need for relevant and accurate information. Particularly, these aspects were consistently also emphasized by the adults interviewed in this study, indicating that these needs persist throughout the life span of LGBTQIA+ individuals. While the study by Steinke et al [36] did not explicitly interview individuals from the community with mental disorder symptoms, it nonetheless underscored the overarching challenge of maintaining mental well-being in the face of the substantial minority stress experienced by these individuals.

In the sample of HCPs, it was noticeable that they predominantly belonged to the SGM group themselves. This raises the question of the extent to which the identity of the treating individuals being perceived as part of the SGM group plays a role. This may establish a “safe space” from the beginning, where the likelihood of experiencing discrimination is reduced, facilitating quicker formation of a trusting therapeutic alliance. This suggests that it could be important to firmly integrate individuals from the target group into the research team. Further research on this topic and its implications for the design of online interventions is needed.

The perspective of HCPs working with SGM individuals with mental health issues, as additionally captured in this study, largely showed consistent results with the perspective of the affected individuals. Interestingly, unlike the SGM individuals with mental health challenges who were interviewed, identity figures in terms of case reports integrated in the online intervention seemed to be of less importance among the HCPs. By contrast, the relevance of attachment and attachment traumas was a higher priority for HCPs than patients. This is consistent with the results of a recent study, which showed that in addition to anxiety symptoms, attachment style, in particular, was a significant predictor of quality of life in the LGBT community, and nonsecure attachment styles were more often found in LGBT people than in heterosexual individuals [37].

The themes highlighted by HCPs partly align with the results of an interview study by Clark et al [38] in which mental health priorities for research were reported, for example, the importance of intimate relationships, stressors specific to SGM status, depression, and support for emotional resilience and well-being.

Regarding the differences between subgroups within the LGBTQIA+ community, only a few points relevant to an online intervention were addressed by the 2 surveyed groups. The intersex group is often neglected and not considered in corresponding offers, while transgender individuals who wish to undergo a gender reassignment surgery frequently face very specific challenges (eg, burdens in the transition process). Nevertheless, the overall intention to develop an intervention for all subgroups was reinforced, as there are many common and connecting elements in the psychological experiences across different subgroups. Therefore, it is crucial for the online intervention to focus on common psychological symptoms rather than subgroup affiliation.

### Strengths and Limitations

To the best of our knowledge, our study is the first to specifically examine the perspectives of SGM adults with mental health challenges and those treating this target group regarding a potential online intervention using a participative approach. The overlapping yet sometimes complementary perspectives overall provide a comprehensive overview from different angles on the aspects that should be integrated into a corresponding online intervention to improve mental health. The interviewed individuals represent a wide spectrum of different sexual and romantic orientations as well as gender identities. A significant strength of this qualitative study is its participatory design, which goes beyond user-centered design and includes end users as active participants in all phases of the research and development process. As recommended, this study used a participatory approach from the outset [39], involving representatives from various German LGBTQIA+ organizations through the LGBTQIA+ advisory board. In addition, perspectives from individuals representing the future target audience (SGM individuals with psychological symptoms) and HCPs working with the target audience were captured in the early development phase of the intervention. Through the collaborative development of the interview guide and planning of study procedures with representatives from various LGBTQIA+ organizations and associations, it was ensured that all important questions were addressed in the interview and that the interviewed individuals felt safe during their participation. Feedback to the study team indicated that all points of contact with the study team during the study were perceived as a safe space for the participants. This sensitive approach was a prerequisite for not reproducing discriminatory experiences within the study but instead creating a secure atmosphere that allowed participants to openly discuss very personal and sometimes deeply distressing experiences during the interviews. By contrast, the collaboration with the LGBTQIA+ advisory board enabled the contextualization of quotes, providing significant added value to the interpretation of the results. Another strength of the study is that we were able to recruit interviewees from different subgroups of the LGBTQIA+ community for the study, which sets us apart from other studies that, for example, often do not take the perspective of intersex people into account [40]. Of course, it cannot be assumed that only one or a few participants speak for the entire subgroup to which they belong, as they can only report on their own personal experiences. Nevertheless, the diverse selection of study participants is an important prerequisite for the diversity of perspectives in the results.

There are also limitations to this study, primarily attributable to its qualitative nature. First, as is typical for qualitative studies, our sample is small and nonrepresentative due to self-selection. However, qualitative studies, which are less limited by resources, should aim for a precalculated larger sample size (eg, using the saturation calculation principle) and more representatives per subgroup, as is the case in this study [41]. Larger studies are necessary to conduct subgroup analyses and potentially highlight the different needs of various subgroups more effectively. Second, the severity of SAD symptoms was not assessed for study inclusion, and no external criteria beyond self-assessment were applied. This approach aimed to include both individuals currently experiencing symptoms and those with a history of such symptoms, allowing reflection on past support needs. However, this limits our ability to determine the actual severity of participants’ symptoms, further compounded by the lack of a formal diagnosis requirement. This decision was made due to the significant gap in undiagnosed LGBTQIA+ individuals who nevertheless experience relevant symptomatology. Therefore, it is important to consider that the SAD symptoms are self-reported when interpreting the data. To minimize the imposition of predefined categories, participants were asked about their self-reported labels for gender identity and sexual orientation. While this approach complicates specific analyses of individual subgroups, it simultaneously emphasizes the individuality of each participant. Intersectionality (belonging to multiple minority groups within the LGBTQIA+ community or based on religion, ethnicity, socioeconomic status, etc) was not explicitly considered in this study, as is the case in many studies on online interventions—a limitation that has been highlighted in a recently published review on the extent of intersectionality in SGM interventions [42]. In agreement with the authors of the aforementioned study [42], we can state that guidelines for clinical practice and evaluation are needed to address intersectionality within interventions for SGM.

### Conclusions

We argue that a queer and intersectional discrimination-sensitive approach to e–mental health care is needed, as the increased risk of psychological distress and illness can be linked to experiences of discrimination and violence based on sexual orientation and gender identity. At the same time, there are several barriers to accessing health care services. Because LGBTQIA+ individuals are a group that is open to digital media and digital media is used for networking and information, digital treatment options are particularly suitable for this population. When developing e–mental health interventions for specific target groups, it is important to include both those immediately affected and professionals working with the population to obtain an intervention that appeals to the target group and is likely to be used. The results of our qualitative participative study serve as a framework for an intervention concept. Besides topics that emerged as crucial for the planned intervention (eg, understanding symptoms in the context of minority stress and specific social challenges and mental needs of LGBTQIA+ individuals), this qualitative study highlighted the strong need for identification figures and the wish for direct interaction with therapists or contact persons within or paralleling the online intervention.

## Appendix

Multimedia Appendix 1 COREQ checklist.

## Abbreviations

CBT: cognitive behavioral therapy
COREQ: Consolidated Criteria for Reporting Qualitative Research
Queer-EMH: Queer-E-Mental Health
HCP: health care professional
LGBT: lesbian, gay, bisexual, and transgender
LGBTQIA+: lesbian, gay, bisexual, transgender, queer or questioning, intersex, asexual, and other sexual orientations and gender identities
SAD: somatoform, anxiety, and depressive
SGM: sexual and gender minority

## Data Availability

The datasets generated or analyzed during this study are available from the corresponding author on reasonable request.
